# Infection of human cytomegalovirus in cultured human gingival tissue

**DOI:** 10.1186/1743-422X-3-84

**Published:** 2006-10-05

**Authors:** Rong Hai, Alice Chu, Hongjian Li, Sean Umamoto, Paul Rider, Fenyong Liu

**Affiliations:** 1 Program in Infectious Diseases and Immunity, Program in Comparative Biochemistry, School of Public Health, 140 Warren Hall, University of California, Berkeley, CA 94720, USA

## Abstract

**Background:**

Human cytomegalovirus (HCMV) infection in the oral cavity plays an important role in its horizontal transmission and in causing viral-associated oral diseases such as gingivitis. However, little is currently known about HCMV pathogenesis in oral mucosa, partially because HCMV infection is primarily limited to human cells and few cultured tissue or animal models are available for studying HCMV infection.

**Results:**

In this report, we studied the infection of HCMV in a cultured gingival tissue model (EpiGingival, MatTek Co.) and investigated whether the cultured tissue can be used to study HCMV infection in the oral mucosa. HCMV replicated in tissues that were infected through the apical surface, achieving a titer of at least 300-fold at 10 days postinfection. Moreover, the virus spread from the apical surface to the basal region and reduced the thickness of the stratum coreum at the apical region. Viral proteins IE1, UL44, and UL99 were expressed in infected tissues, a characteristic of HCMV lytic replication in vivo. Studies of a collection of eight viral mutants provide the first direct evidence that a mutant with a deletion of open reading frame US18 is deficient in growth in the tissues, suggesting that HCMV encodes specific determinants for its infection in oral mucosa. Treatment by ganciclovir abolished viral growth in the infected tissues.

**Conclusion:**

These results suggest that the cultured gingival mucosa can be used as a tissue model for studying HCMV infection and for screening antivirals to block viral replication and transmission in the oral cavity.

## Background

Human cytomegalovirus (HCMV) is a ubiquitous herpesvirus that causes mild or subclinical diseases in immunocompetent adults but may lead to severe morbidity and mortality in neonates and immunocompromised individuals [1,2]. For example, disseminated HCMV infection, common in AIDS patients and organ transplant recipients, is usually associated with gastroenteritis, pneumonia, and retinitis [3,4]. Moreover, HCMV is one of the leading causes of birth defects and mental retardation in newborns [5,6]. Understanding the biology of CMV infection and developing novel anti-CMV approaches are central in the treatment and prevention of CMV-associated diseases.

HCMV infection in the oral cavity plays an important role in its pathogenesis and transmission. HCMV is among the most common causes of oral diseases associated with AIDS patients [7,8]. Active viral replication in the oral tissue induces CMV-associated oral manifestations such as ulcerations, aphthous stomatitis, necrotizing gingivitis, and acute periodontal infection [9-13]. Persistent and latent infections have also been found in oral tissues. The presence of infectious particles in the oral cavity including saliva is believed to be a major source of HCMV horizontal transmission [1,6]. Indeed, initial infection of the oral mucosa by HCMV, primarily through casual contact, is believed to be one of the major routes of horizontal transmission among individuals, and the consequent viral replication and spread in oral tissues leads to the establishment of lifelong latent infection. Elucidating the mechanism of HCMV infection in the oral mucosa and blocking viral replication in infected oral tissues are essential for the treatment and prevention of CMV transmission and systemic infections.

HCMV belongs to the β family of herpesviruses and contains a linear 230 kb double-stranded DNA genome that is predicted to encode more than 200 proteins [14,15]. There are currently few animal models available to study HCMV infection and pathogenesis and to determine efficacy of various antiviral therapies. This is largely due to the fact that HCMV infection and replication are limited to human cells [1,2]. Consequently, little is known about the mechanism of viral pathogenesis, such as how HCMV infects the oral mucosa.

One of the most powerful approaches to study viral pathogenesis is to develop a cultured tissue model that can mimic natural infection in human tissues in vivo. The SCID-hu mouse, in which different fetal human tissues are implanted into the kidney capsule of a severe combined immunodeficient (SCID) mouse, has been shown to be a useful model to study HCMV replication and to screen antiviral compounds in human tissues [16,17]. In these animals, the implanted human fetal tissues continue to grow and differentiate. HCMV was directly inoculated into the implanted tissues and viral replication was monitored. SCID-hu mice implanted with different human tissues from the liver, thymus, bone, retina, and skin have been shown to support HCMV replication and can be used as models to study HCMV infection in these human tissues in vivo [16,18]. However, the difficulty in generating these animals limits the use of the models. Furthermore, the use of fetal tissues in SCID mice presents a challenge to study HCMV infection in adult tissues, such as in the oral mucosa, because the implanted tissues need to differentiate properly into adult tissues in the mouse microenvironment. Currently, no SCID mice with human oral mucosa implants have been reported.

Recently, three-dimensional models of the human oral epithelia that exhibit a buccal or gingival phenotype, such as EpiGingival from MatTek, Co., have been developed [19-22]. In these models, normal human keratinocytes are differentiated into tissues in serum free media. The gingival model has 10–20 layers of viable, nucleated cells and is partially cornified at the apical surface. These models exhibit very similar histological characteristics to human oral tissues in vivo. Thus, they can serve as a tissue model for human oral epithelia, such as gingival mucosa, and can potentially be used to study oral physiology and transmission of infectious pathogens.

The development of reconstructed tissues of human oral cavity provides an invaluable cultured tissue system for studying the biology of CMV infection. To study the function of viral-encoded genes in supporting HCMV infection, we can generate a collection of viral mutants by introducing mutations into the viral genome and screening viral mutants in both cultured cells and tissues for potential growth defects [23]. The construction of HCMV mutants has been reported using site-directed homologous recombination and cosmid libraries of overlapping viral DNA fragments, and recently, using a bacterial artificial chromosome (BAC)-based approach [24-30]. Examining the growth of these mutants in the oral tissue model should facilitate the identification of viral genes responsible for HCMV tropism in the oral mucosa and for transmission. Furthermore, the tissue model can be used for screening antiviral compounds and for developing novel strategies for preventing HCMV infection in oral cavity and its transmission among human populations.

In this study, we examined the infection of HCMV in a cultured gingival mucosa model (EpiGingival, MatTek Co.) and determined whether the cultured tissue is suitable to study HCMV infection in vivo. Both laboratory-adapted viral strain and low-passaged clinical isolate were shown to infect the human tissue via the apical surface. Investigation of the growth of these viruses indicates that the viral strains replicate at a similar level, reaching a 300-fold higher titer after 10 days post infection. Histological examination of tissues infected via the apical surface indicated that these viruses spread from the apical surface to the suprabasal region. Moreover, Western analyses demonstrated the expression of viral proteins IE1, UL44, and UL99 in the infected tissues, suggesting that the infection process represents a classic lytic replication that is associated with primary HCMV infection in vivo. Growth studies of a collection of eight viral mutants indicated that a mutant with deletion at open reading frame US18 is deficient in growth in human oral tissues. Treatment of infected tissues with ganciclovir, which is effective for anti-HCMV therapy in vivo [31,32], abolished viral growth in the cultured tissues. These results provide the first direct evidence that the cultured gingival mucosa is an excellent tissue model for studying HCMV infection in vivo and for screening antiviral compounds to block HCMV infection and transmission in the oral cavity.

## Results

### Growth of different HCMV strains in cultured human oral tissue

**Figure 1:**
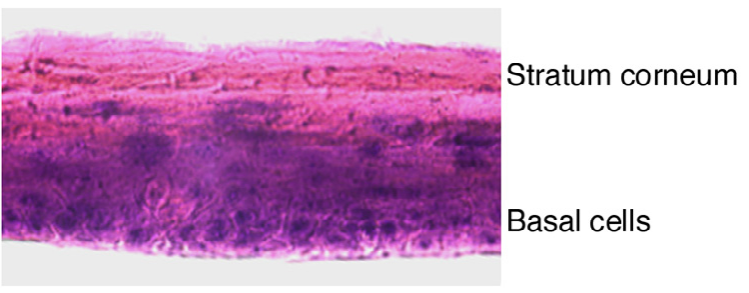
Hematoxylin and eosin staining of EpiGingival tissues (magnification, ×400). Upon arrival, the tissues were cultured for 12 hours prior to viral infection, fixed with Streck Tissue Fixative, frozen in 2-methylbutane submerged in liquid nitrogen, cross-sectioned at 9 μm using a LEICA cryostat LC1900 sectioner, stained with hematoxylin and eosin, and visualized with a Nikon TE300 microscope.

The MatTek gingival tissue model (EpiGingival) contains normal human oral keratinocytes cultured in serum-free medium to form three-dimensional differentiated tissues. Hematoxylin and eosin staining of tissue cross-sections indicates that the cultured tissue shows an architecture very similar to human gingival mucosa in vivo (Figure 1, see Figure 4A) [22]. The cultured tissue is 10–20 cell layers thick and consists of a cornified apical surface and a non-cornified basal region (Figure 1). The thickness and morphology of the apical stratum corneum and the basal cell layers are similar to those in the gingival tissues in vivo. As observed in vivo, cells at the basal region of the cultured tissue continue to divide and differentiate, and apical surface cells continue to cornify to form the stratum corneum. Furthermore, immunohistochemical staining indicates that distributions of different cytokeratins (e.g. K13 and K14) in cultured tissues are like those found in vivo [22,33] (data not shown). Thus, the cultured tissue exhibits characteristics in structure (thickness, morphology, and organization), cell type and differentiation, and protein expression and composition as observed in vivo, and can be a model representing the oral tissue [22].

**Figure 2:**
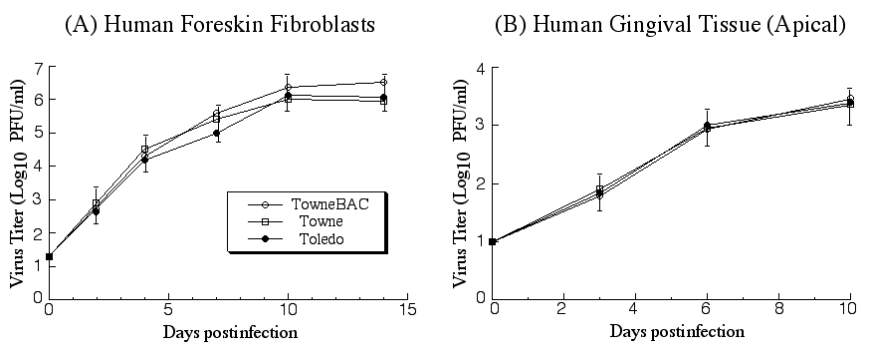
Growth of different HCMV strains (Toledo, Towne, and Towne_BAC_) in cultured cells (A) and cultured gingival tissues (B). In (A), human foreskin fibroblasts (HFFs) (1 × 10^6 ^cells) were infected with each virus at a MOI of 0.05. At 0, 2, 4, 7, 10, and 14 days post infection, cells and culture media were harvested and sonicated. In (B), the tissues were infected with 2 × 10^4 ^PFU of each virus at the apical surface of the tissue. At 0, 3, 6, and 10 days post infection, the tissues were harvested, suspended in a small volume of 10% milk, and sonicated. The viral titers were determined by plaque assays on HFFs. The limit of detection was 10 PFU/ml of the tissue homogenate. The values of the viral titer represent the average obtained from triplicate experiments. The standard deviation is indicated by the error bars.

To determine whether the cultured tissues are permissive to HCMV infection and replication, two different HCMV strains (Towne and Toledo) and a mutant (Towne_BAC_), were used in our initial experiments. Towne is a laboratory-adopted strain that has been passaged many times in vitro in human fibroblasts; whereas Toledo is an HCMV clinical isolate passaged in limited numbers in vitro [34,35]. Towne_BAC _was derived from Towne by inserting a bacterial artificial chromosome (BAC) sequence into the viral genome and replacing the dispensable, 10 kb US1-US12 region [36]. The Towne_BAC _DNA, while maintained as a BAC-based plasmid in *E. coli*, produces infectious progeny in human fibroblasts and retains a wild type-like growth characteristic *in vitro *(Figure 2A) [23,36]. Each of these viruses was used to infect the tissues by inoculating at the apical surface with 2 × 10^4 ^PFU. The infection through the apical surface serves as a model for HCMV infection via gingival mucosa surface. The infection was carried out for 10 days. We observed that the structure of the tissue remained intact up to 10 days in culture and started to disintegrate after 12 days incubation (data not shown). At different time points post infection, the tissues were harvested and the titers of the viruses were determined. The viral strains were able to grow in the tissues since viral titers increased by at least 300-fold during a 10 day infection period (Figure 2B). Thus, the gingival tissues support active HCMV lytic replication. No differences in growth among these viruses were found, suggesting that the lab-adopted Towne strain and its derivative, Towne_BAC_, grow as well as the clinical low-passaged Toledo strain. In subsequent experiments, Towne_BAC _was used as an HCMV representative to study viral infection in the gingival tissues. This mutant contains the gene coding for green fluorescence protein (GFP) and therefore, infection can be easily monitored in the tissues by detecting GFP expression [23,36].

### Viral protein expression and histological changes in cultured human oral tissue upon HCMV infection

**Figure 3:**
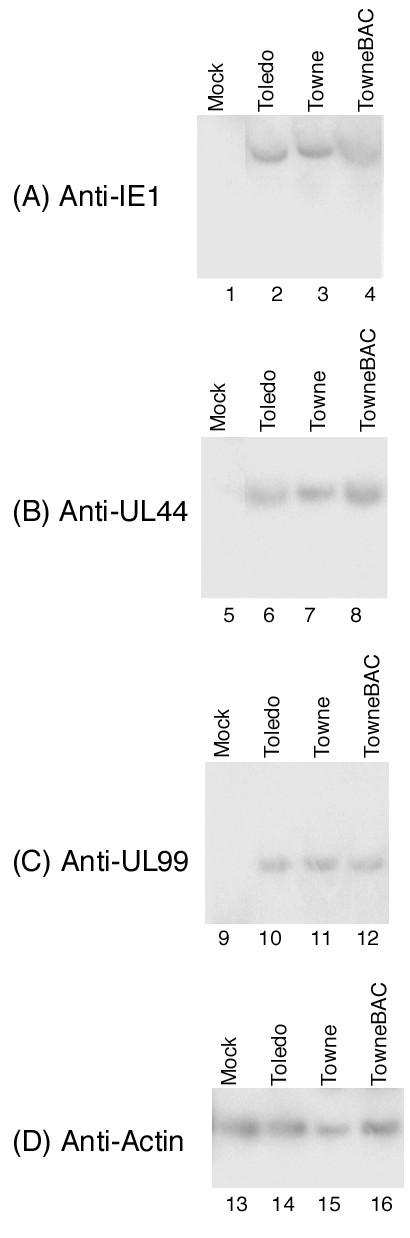
Expression of HCMV lytic proteins as determined by Western blot analysis. Protein samples were isolated from the cultured EpiGingival tissues that were either mock-infected (lanes 1, 5, 9, and 13) or infected with HCMV (2 × 10^4 ^PFU) (lanes 2–4, 6–8, 10–12, and 14–16) for 6 days, separated in SDS-polyacrylamide gels, and then transferred to membranes. One membrane was allowed to react with a monoclonal antibody (Anti-actin) against human actin (D) while the others were stained with the antibodies (Anti-IE1, Anti-UL44, and Anti-UL99) against HCMV IE1, UL44, or UL99, respectively (A-C). The expression of human actin was used as the internal control for the quantitation of the expression of HCMV proteins.

HCMV oral transmission begins when the virus enters the mucosal (apical) surface of oral tissues (e.g. gingival tissues), replicates in the surface cell layers, and spreads to neighboring cells and tissues in the basal regions [1,7]. To determine whether HCMV infection of the MatTek gingival tissues can be a model for viral infection in vivo, two sets of experiments were carried out. First, Western analysis was used to determine whether viral lytic proteins were expressed, as observed in productive HCMV infection in vivo. Tissues were infected with 2 × 10^4 ^PFU of either HCMV Toledo, Towne, or Towne_BAC _strains. Protein extracts were isolated from tissues that were either mock-infected or infected with HCMV at 6 days post infection. Viral proteins were separated electrophoretically in SDS-polyacrylamide gels and electrically transferred to identical membranes. One of the membranes was stained with monoclonal antibody against human actin (anti-actin) (Figure 3D) and the other membranes were stained with monoclonal antibodies against viral IE1, UL44, and UL99 proteins (Figure 3A–C). The expression of actin serves as an internal control for the quantitation of HCMV protein expression in the tissues. IE1 is a viral immediate-early (α) protein, while UL44 and UL99 encode viral early (β) and late (γ) proteins, respectively [2]. These proteins serve as the representatives for the expression of viral α, β, and γ genes. As shown in Figure 3, IE1, UL44 and UL99 were expressed in infected tissues. Combined with the growth analysis (Figure 2), these results indicate that the cultured tissues are permissive to HCMV infection and can support viral lytic gene expression and replication.

In the second set of experiments, infection of these tissues was studied using both conventional histological and fluorescent microscopy. Two different staining methods were employed. First, tissues were stained with hematoxylin and eosin in order to examine their structures. Second, since Towne_BAC _contains a GFP expression cassette [36], fluorescent microscopy was used to detect GFP expression and to visualize infected cells.

**Figure 4:**
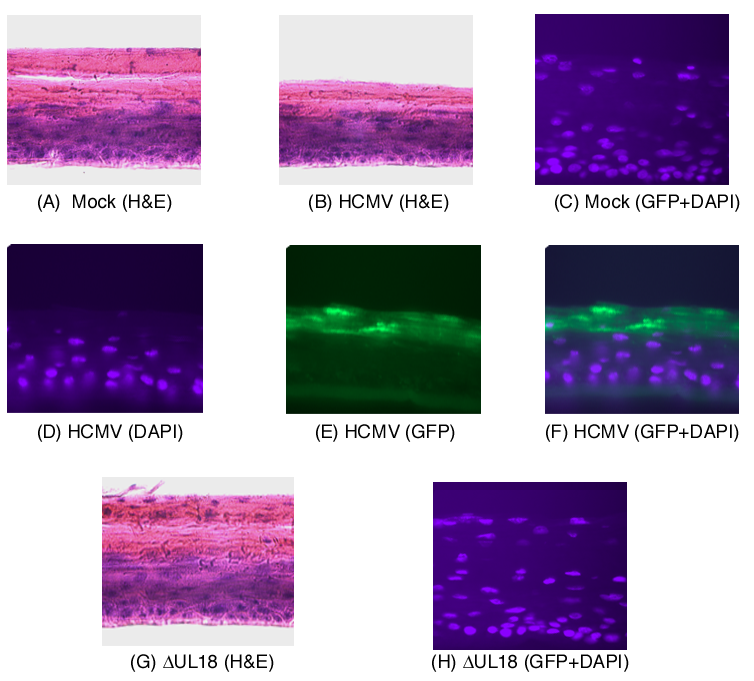
Hematoxylin/eosin (A-B and G) and fluorescent staining (C-F, H) of EpiGingival tissues. The tissues were either mock infected (A, C) or infected with 2 × 10^4 ^PFU of HCMV mutant ΔUS18 (G and H) and the parental Towne_BAC _(B, D, E, and F), harvested at 7 days post infection, fixed with Streck Tissue Fixative, frozen in 2-methylbutane submerged in liquid nitrogen, and cross-sectioned at 9 μm using a LEICA cryostat LC1900 sectioner, stained with either hematoxylin/eosin or DAPI, and visualized (magnification, ×400). The cells that were infected with Towne_BAC _and ΔUS18, which carried a GFP expression cassette, were visualized by detecting the expression of GFP (C, E, F, and H). The images of the DAPI-staining tissues (DAPI) (D) and the infected cells that expressed the GFP (GFP) (E) were used to generate the composite image (GFP+DAPI) (F). Similar composite images (GFP+DAPI) are shown in (C) and (H).

As shown in Figure 4, mock-infected tissues maintained the characteristic gingival mucosal structure during the infection period. In these tissues, the cells at the basal surface continue to divide while those at the apical surface differentiate and cornify, forming a characteristic stratum corneum (Figure 4A). In the tissues that were infected through the apical surface, GFP staining was found in the cells near the apical surface, suggesting that the apical cells were infected with HCMV (Figure 4C–F). Compared to mock-infected tissues, the thickness of the stratum corneum in the infected tissues was significantly reduced (Figure 4B), possibly because the active replication of HCMV in apical cells induces cellular lysis and disrupts cellular differentiation and generation of the stratum corneum. Active HCMV replication in the apical surface has been observed in vivo and is associated with reduced thickness and destruction of the oral epithelial surface [1,9,11]. Thus, our results suggest that HCMV infection of cultured gingival tissues via the apical surface corresponds to its pathogenesis in vivo.

### Deficient growth of HCMV mutants in infected human oral tissues

The ability of HCMV to infect and replicate in cells of the oral cavity is responsible for its pathogenesis in the oral mucosa, including viral-associated gingivitis and oral lesions. However, little is currently known about the mechanism of how HCMV is able to infect and replicate in oral tissues. Equally elusive is the identity of viral determinants responsible for oral infection. Specifically, it is unknown whether HCMV encodes specific genes responsible for its infection in the gingival mucosa. Through the use of a BAC-based mutagenesis approach, we have recently generated a library of HCMV mutants containing deletions in each open reading frame (ORF) [23]. If a viral ORF is essential for viral infection in the oral tissue, the corresponding mutant with the deletion of the ORF is expected to be deficient in infecting and replicating in the tissue. Using the gingival tissue as the model, several experiments were performed to determine whether viral mutants that are attenuated in growth in the oral mucosa can be identified.

**Figure 5:**
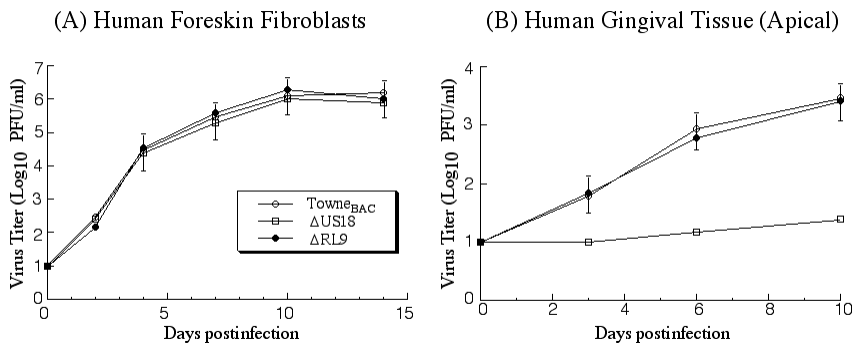
Growth of HCMV mutants ΔUS18, ΔRL9, and the parental Towne_BAC _in cultured cells (A) and gingival tissues (B). In (A), human foreskin fibroblasts (HFFs) (1 × 10^6 ^cells) were infected with each virus at a MOI of 0.05. At 0, 2, 4, 7, 10, and 14 days post infection, cells and culture media were harvested and sonicated. In (B), the tissues were infected with 2 × 10^4 ^PFU of each virus at the apical surface of the tissue. At 0, 3, 6, and 10 days post infection, the tissues were harvested, suspended in 10% milk, and sonicated. The viral titers were determined by plaque assays on HFFs. The limit of detection was 10 PFU/ml of the tissue homogenate. The values of the viral titer represent the average obtained from triplicate experiments. The standard deviation is indicated by the error bars.

A collection of eight different mutants was used in our initial screen (Table 1). Each mutant was derived from Towne_BAC _and contains a deletion in ORF UL13, UL24, UL25, UL108, US18, US20, US29, or RL9, respectively [23]. In these mutants, the deleted ORF sequence was replaced with a kanamycin-resistance gene (KAN) expression cassette, which provides antibiotic resistance for rapid selection and isolation of the bacteria carrying the mutated Towne_BAC _sequence. All mutants grew as well as the parental Towne_BAC _in primary human foreskin fibroblasts (HFFs), suggesting that these ORFs are not essential for viral replication in vitro in cultured fibroblasts (Table I and Figure 5A). The functions of many of these deleted ORFs are currently unknown. However, they are present in all HCMV strains whose sequences have been determined [14,15,23,37,38]. Hence, these genes may play an important role in HCMV infection in vivo, such as in viral transmission and infection in the oral cavity.

**Table 1:** Comparison of the growth properties of eight different mutants in primary human foreskin fibroblasts (HFFs) and cultured human gingival tissues (Gingival tissues) with those of the parental Towne_BAC_.

**Mutants**	**HFFs**	**Gingival Tissue**
Towne_BAC_	+++	+++
ΔUL13	+++	++
ΔUL24	+++	+++
ΔUL25	+++	+++
ΔUL108	+++	+++
ΔUS18	+++	+
ΔUS20	+++	+++
ΔUS29	+++	+++
ΔRL9	+++	+++

Cells (1 × 10^6 ^HFFs) or tissues were infected with each virus at 2 × 10^4 ^PFU and at 10 days post-infection, cells and tissues were harvested and sonicated. The viral titers were determined by plaque assays on HFFs in triplicate experiments [46]. +++, titer similar to that of Towne_BAC_; ++, titer about 10 times lower than that of Towne_BAC_; +, titer at least 100 times lower than that of Towne_BAC_.

To determine whether any of these HCMV mutants are deficient in growth and infection in cultured gingival tissues, the tissues were infected via the apical mucosal surface with each viral mutant at an inoculum of 2 × 10^4 ^PFU. Infected tissues were harvested at 10 days post infection and viral titers in the tissues were determined. The titers of mutant ΔUS18 and ΔUL13 at 10 days post infection were approximately 100- and 10- folds lower than those of the parental Towne_BAC_, respectively, while other mutants, such as ΔUL24 and ΔRL9, replicated as well as the parental virus (Table I and Figure 5B). Thus, mutants ΔUL13 and ΔUS18 appeared to be deficient in infecting the tissues via the apical surface. Both ΔUL13 and ΔUS18 were derived from the parental Towne_BAC _by replacing the UL13 and US18 ORFs, respectively, with a DNA sequence (KAN) that confers antibiotic resistance to kanamycin in *E. coli *[23]. Because ΔRL9 replicates as well as the parental Towne_BAC _(Figure 5), the presence of the KAN cassette in the viral genome per se does not significantly affect the ability of the virus to grow in the tissues. Thus, these results suggest that the growth defect of ΔUS18 may be due to the deletion of the US18 ORF.

**Figure 6:**
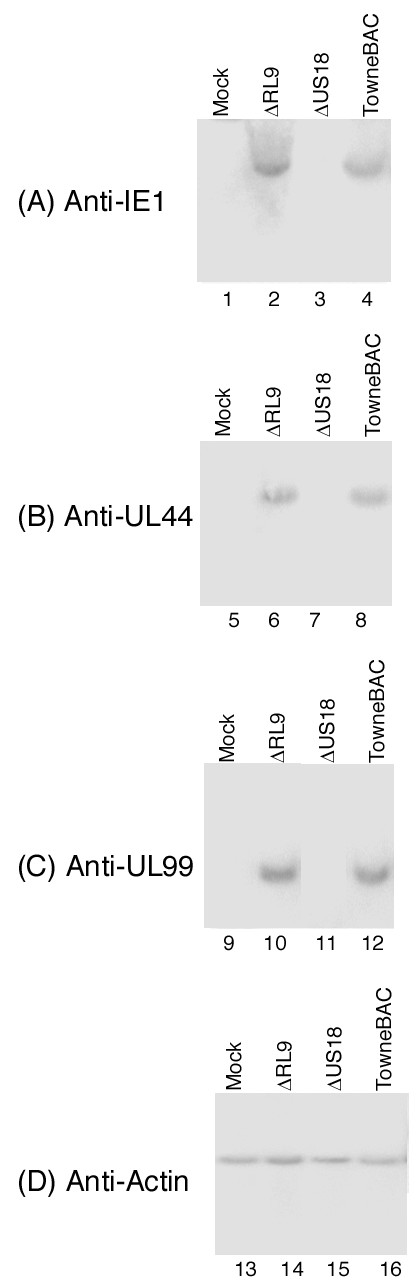
Expression of HCMV lytic proteins as determined by Western blot analysis. Protein samples were isolated from the cultured EpiGingival tissues that were either mock-infected (lanes 1, 5, 9, and 13) or infected with HCMV (2 × 10^4 ^PFU) (lanes 2–4, 6–8, 10–12, and 14–16) for 72 hours, separated in SDS-polyacrylamide gels, and then transferred to membranes. One membrane was allowed to react with a monoclonal antibody (Anti-actin) against human actin (D) while the others were stained with the antibodies (Anti-IE1, Anti-UL44, and Anti-UL99) against HCMV IE1, UL44, or UL99, respectively (A-C).

Two series of experiments were further carried out to study how ΔUS18 is defective in growth in the cultured tissues. First, viral infection in the tissues was studied by examining hematoxylin and eosin-stained tissues and visualizing GFP expression in infected cells. At 7 days post infection, the structure of the apical region in the ΔUS18-infected tissues was similar to that of uninfected tissues, and the thickness of the stratum corneum was not reduced as observed in the Towne_BAC_-infected tissues (Figure 4G–H). Little GFP staining was found in the ΔUS18-infected tissues (Figure 4H) while substantial levels of GFP staining were detected in tissues infected with ΔRL9 and Towne_BAC _(Figure 4E–F, data not shown). These observations support the growth analysis results (Figure 5) and show that ΔUS18 is deficient in infection and replication in gingival tissues. Second, Western analyses were used to examine the expression of viral proteins. As shown in Figure 6, at 72 hours post infection, the expression levels of IE1, UL44, and UL99 in ΔUS18-infected tissues were minimal and significantly lower than those in Towne_BAC_-infected tissues. Thus, the infection of ΔUS18 appeared to be blocked prior to or at viral immediate-early gene expression, probably during viral entry, decoating, or transporting the capsid to the nuclei. Because similar levels of these proteins were found in tissues that were infected with ΔRL9 and Towne_BAC _(Figure 6), the presence of the KAN cassette in the viral genome (e.g. ΔRL9) per se does not significantly affect viral protein expression in the tissues. These observations suggest that the defect in protein expression of ΔUS18 may be due to the deletion of the US18 ORF.

### Inhibition of HCMV growth in human oral tissues after ganciclovir treatment

**Figure 7:**
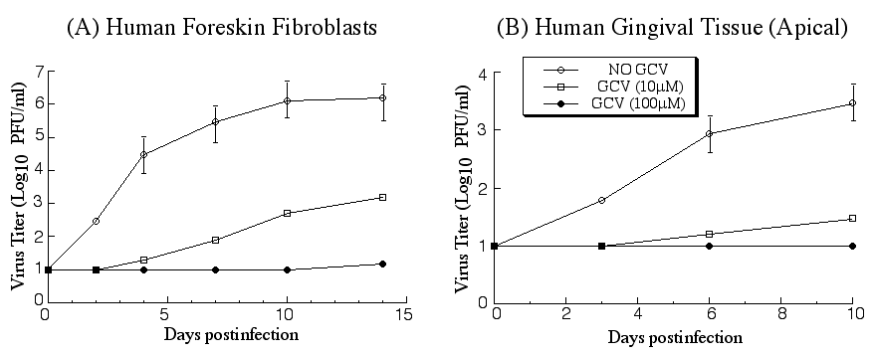
Growth of HCMV in cultured cells (A) and gingival tissues (B) that were treated with different concentrations of ganciclovir. In (A), human foreskin fibroblasts (HFFs) (1 × 10^6 ^cells) were infected with each virus at a MOI of 0.05. At 0, 2, 4, 7, 10, and 14 days post infection, cells and culture media were harvested and sonicated. In (B), the tissues were infected with 2 × 10^4 ^PFU of Towne_BAC _at the apical surface of the tissue. At 0, 3, 6, and 10 days post infection, the tissues were harvested, suspended in 10% milk, and sonicated. Different concentrations (10 μM or 100 μM) of GCV were added to the cultured media at 24 hours post infection. The viral titers were determined by plaque assays on HFFs. The limit of detection was 10 PFU/ml of the tissue homogenate. The values of the viral titer represent the average obtained from triplicate experiments. The standard deviation is indicated by the error bars.

One of our objectives is to establish an in vitro cultured tissue model to screen antiviral compounds and determine their potency in inhibiting HCMV growth and replication in human oral tissue. To determine the feasibility of using the gingival tissue for antiviral compound screening and testing, two sets of experiments were carried out using ganciclovir, which functions as a nucleoside analog and is effective in treating HCMV infection in vivo by blocking viral DNA replication [31,32]. In the first set of experiment, oral tissues were treated with different concentrations of ganciclovir for 4 hours prior to viral infection. In the second set of experiments, tissues were infected with Towne_BAC _for 24 hours and then treated with different concentrations of ganciclovir. The tissues were harvested at different time points post infection and the growth of HCMV was assayed by determining the viral titers. Treatment of ganciclovir reduced the growth of HCMV in HFFs (Figure 7A) [31,32]. Significant inhibition of HCMV growth was also observed in the gingival tissues when ganciclovir was added 24 hours after viral infection (Figure 7B). Similar levels of inhibition of viral growth in the tissues were found when the tissues were incubated with the drug before viral infection (data not shown). Previous studies have shown that treatment of ganciclovir blocks HCMV infection in cultured fibroblasts regardless whether the drug was added before or 24 hours after viral infection [31,32]. These results strongly suggest that cultured gingival tissues can be a suitable model for screening and testing antiviral compounds for inhibiting HCMV growth and replication.

## Discussion

The oral mucosal epithelia represent one of the most common sites encountered with microbial organisms for infection and transmission [39-41]. Both commensal (nonpathogenic) and pathogenic bacteria and yeast have been found in the epithelia [39,40]. The mucosa surface also appears to be susceptible to infection by a variety of viruses including HCMV, herpes simplex virus, HIV, and human papillomavirus [7,41]. The development of human reconstructed tissues of the oral cavity that exhibit the differentiated characteristics found in vivo will provide excellent research tools to study the biology of infections by these pathogens, to screen antimicrobial compounds, and to develop therapies against oral diseases associated with these infections.

HCMV primarily propagates and replicates in human cells, and there are few animal models available to study HCMV infection and pathogenesis [1,2]. Little is known whether cultured human oral tissues can support HCMV lytic replication in vitro and be used to study HCMV infection. In this study, we have characterized the infection of HCMV in a cultured gingival tissue model. Several lines of evidence presented in this study strongly suggest that the cultured oral tissues support HCMV replication, and can be used as a model for studying HCMV pathogenesis, screening antivirals, and developing therapies for treating CMV infections in the oral cavity. First, the cultured tissue morphology and architecture used in our experiments was histologically similar to that found in vivo (Figure 1). Tissue structure remained intact for up to 10 days in the uninfected tissues. Hematoxylin and eosin staining showed no significant changes in tissue structure, except increased cornification and cell proliferation toward the apical surface (Figure 4A). These results suggest that our cultured conditions do not significantly affect the continuous differentiation and growth of the tissues and that the tissues exhibit similar characteristics found in vivo.

Second, both laboratory-adapted "high passage" Towne strain and clinical "low passage" Toledo strain were able to infect the apical surface and establish productive infection (Figure 2). An increase of at least 300-fold in viral titers was found in the infected tissues after a 10-day infection period. Thus, HCMV can replicate in the cultured tissue as it does in vivo in oral tissues.

Third, viral lytic proteins, IE1, UL44, and UL99, were detected in cultured tissues (Figure 3). These proteins are commonly found in infected tissues in vivo, with IE1, UL44, and UL99 expressed at the immediate-early, early, and late stage of the HCMV lytic replication cycle, respectively [2]. These results suggest that HCMV infection in the cultured tissues exhibits similar gene and protein expression profiles as found in vivo.

Fourth, fluorescence microscopy experiments indicated that HCMV can spread within the cultured tissue as observed in vivo (Figure 4). Towne_BAC_, which carries a GFP expression cassette and a BAC sequence [36], was used in our experiments. Viral infection and spread can be monitored by detecting the GFP expression. HCMV spread started from the apical surface, the inoculation site, to the suprabasal regions in the tissues. Initial viral infection at the apical surface and subsequent spread to the suprabasal region have been observed in oral mucosa in vivo and are believed to represent a common route for viral transmission among casual contacts [1]. Active HCMV replication led to lysis of infected cells, damage of tissues, and reduced thickness of the cornified cell layers in the cultured oral tissues (Figure 4). Similar observations are found in vivo, as uncontrolled replication of HCMV leads to lesions and ulcers in the oral epithelia [1,9,11]. Thus, HCMV infection in cultured oral tissues appears to cause similar cytopathic effects and pathological changes as found in vivo.

Fifth, treatment with ganciclovir, which is effective in treating HCMV infection in vivo [31,32], abolished the growth of HCMV in cultured tissues (Figure 7). These results indicate that the cultured tissue model can be used for screening antiviral compounds for blocking HCMV infection and replication in the oral cavity.

The availability of a cultured oral mucosa model will provide a unique opportunity to study HCMV pathogenesis in oral tissues and to identify viral determinants responsible for HCMV infection in oral cavity. We have initiated a series of experiments to use the cultured tissues to screen a pool of viral mutants with deletions in different HCMV ORFs (Table 1). ΔUS18 was found to be defective in growth in the cultured tissues (Figure 5). These observations suggest that HCMV encodes specific determinants for its infection and replication in the oral mucosa. Moreover, these results validate the use of the cultured tissue as a model for identifying viral genes important for oral infection and for studying the mechanism of how HCMV replicates and causes viral-associated diseases in oral cavity.

The function of US18 is currently unknown. US18 is only found in the HCMV genome and no sequence homologues are found in other human herpesviruses or rodent CMVs (e.g. murine CMV (MCMV)) [14,15,38]. It is believed that some genes from a particular CMV (e.g. HCMV) might have co-evolved with its respective host and interacted with specific components of the host and therefore, are unique and may not share significant sequence homologies with CMVs from other species (e.g. MCMV). For example, US11 and US28, which are dispensable for HCMV replication in vitro, function to down-regulate the major histocompatibility complex (MHC) class I molecules and stimulate vascular smooth muscle cell migration, respectively [42,43]. While little is known about CMV determinants important for viral infection in the oral mucosa, previous studies have shown that salivary gland gene 1 (sgg1), a gene that is unique to MCMV and is dispensable for viral replication in vitro, is important for MCMV infection in salivary glands [44]. Likewise, the function of US18 may be involved in species-specific interactions between HCMV and humans, such as the potential interactions in the apical surface of oral epithelia. Like US11 and US28, US18 is dispensable for HCMV replication in vitro since ΔUS18 grows as well as the parental Towne_BAC _in human fibroblasts (Figure 5A). US18 has been predicted to encode a membrane protein [14,15,38] and is found to be expressed predominantly in the cytoplasm [45]. Our results of Western analysis and examination of the ΔUS18-infected tissues (Figures 4 and 6) suggest that the infection of ΔUS18 is very limited and may be blocked prior to or at the step of viral immediate-early gene expression, possibly during viral entry, decoating, or transporting the capsids to the nuclei.

To confirm the assignment of functionality of a particular viral gene (e.g. US18), it is probably necessary to restore the mutation back to the wild type sequence and determine whether the phenotype of the rescuant viruses is similar to that of the parental virus. However, the rescue procedures may potentially introduce adventitious mutations that occur elsewhere in the genome. Meanwhile, it is possible that the deletion of a target ORF (e.g. US18) might affect the expression of other viral genes, including those in nearby regions, as the deleted region may function as a regulatory element important for the expression of these genes, in addition to encoding the target ORF. Extensive studies are needed to demonstrate that the deletion does not affect any other gene expression in the viral genome. Alternatively, a viral mutant that contains a subtle mutation, such as point mutations, to inactivate the ORF can be generated. Examination of the phenotype of this second isolate should confirm the results obtained from the first mutant. Further characterization of these mutants and the genes mutated will identify the HCMV determinants important for viral pathogenesis and elucidate the functional roles of these ORFs in HCMV infection.

Our results demonstrate that the cultured tissues provide a useful system to study HCMV pathogenesis and to identify viral determinants responsible for HCMV infection in oral cavity. However, fully differentiated gingival tissues currently can be maintained in vitro for only a very limited period of time (~10–14 days). In our experience, after 11 days of culture upon arrival, the tissues began to deteriorate and their structures and morphologies changed (data not shown). Thus, the cultured tissues currently can only be used to study HCMV lytic but not latent infection. Further studies, such as tissue engineering and improving culture conditions and media compositions, will facilitate the development of this exciting model to study oral biology and infections. Investigation of HCMV infection and characterization of different viral strains and mutants in these cultured tissues will provide valuable insight into the mechanism of how HCMV infects oral epithelia, achieves successful transmission, and causes viral-associated oral complications. Furthermore, these results will facilitate the development of new compounds and novel strategies for treating CMV-associated oral lesions and preventing viral transmission.

## Conclusion

In this report, we investigated the infection of HCMV in a cultured gingival tissue model and determined whether the cultured tissue can be used to study HCMV infection in the oral mucosa. HCMV replicated in the cultured tissues that were infected through the apical surface, spread from the apical surface to the basal region, and reduced the thickness of the stratum coreum at the apical region. Our results that a mutant with a deletion of open reading frame US18 is deficient in growth in the tissues provided the first direct evidence to suggest that HCMV encodes specific determinants for its infection in gingival tissues. Viral infection in these tissues resembled HCMV lytic replication observed in vivo and was inhibited by treatment of ganciclovir. These results suggest that the cultured gingival tissue can be used as a cultured human tissue model for studying HCMV infection and for screening antivirals to block viral replication and transmission in the oral cavity.

## Methods

### Viruses and cells

Primary human foreskin fibroblasts (HFFs) (CC-2509) from Clonetics (San Diego, CA) were cultured in a humidified incubator at 37°C and in the presence of 5% CO_2_. Cells were maintained in Dulbecco's modified Eagle medium (DMEM) supplemented with 10% (vol/vol) fetal bovine serum (GIBCO/BRL), 1% (vol/vol) penicillin-streptomycin (GIBCO/BRL), and 0.2% (vol/vol) fungizone amphotericin B (GIBCO/BRL) [23]. The HCMV Towne strain was obtained from the American Type Culture Collection (ATCC, Rockville, MD). The Toledo strain was a gift from Dr. Edward Mocarski (Stanford University) [16,35]. Towne_BAC _and all the mutant viruses used in this study have been described previously [23,36] and were propagated in HFFs.

### Viral infection of human tissue

Human gingival tissues (EpiGingival), obtained from MatTek Co (Ashland, MA), are living reconstructed oral epithelial tissues of 10–20 layers of cells that are derived from human primary oral keratinocytes and allowed to differentiate to a structure characteristic to that in vivo [22]. The tissues arrived in Millipore Millicell CM culture insert wells and were approximately 0.1 mm thick and 9 mm in diameter. After overnight refrigeration (4°C, manufacturer's recommendations), the tissues were equilibrated by transferring them to 6 well plates containing 5 ml of assay media (MatTek Co.) per well and incubated at 37°C and 5% CO_2 _for 1 hour. A small volume of 2 × 10^4 ^PFU HCMV (0.1~0.2 ml) was then directly added to the apical surface of the tissues. After incubation with the viral inoculum at 37°C and 5% CO_2 _for 4 hours, the tissues were washed to remove the inoculum. The tissues were replenished with fresh serum-free media containing growth factors every 48 hours. At different time points post infection, the tissues were collected and processed for determination of viral titers and for histochemical and fluorescent microscopy analysis.

### Analysis of the growth of viruses in human oral tissues

The tissues were suspended in a small volume of 10% skim milk, followed by sonication. The tissue homogenates were titered for viral growth on HFFs in 6-well tissue culture plates (Corning Inc., Corning, NY) [23]. Cells were inoculated with 1 ml of the sonicated tissues in 10-fold serial dilutions. After two hours of incubation at 37°C and 5% CO_2_, cells were washed with complete media, overlaid with fresh complete medium containing 1% agarose, and cultured for 7–10 days. Plaques were counted under an inverted microscope. Each sample was titered in triplicate and viral titers were recorded as PFU/ml of tissue homogenates. The limit of virus detection in the tissue homogenates was 10 PFU/ml of the sonicated mixture. Those samples that were negative at a 10^-1 ^dilution were designated a titer value of 10 (10^1^) PFU/ml.

### Tissue preparation and processing for histological studies

Human oral tissues were fixed in Streck Tissue Fixative (Streck Laboratories, La Vista, NE) and then placed in 30% sucrose overnight. To prepare for cryostat sectioning, tissues were embedded in Histo Prep (Fisher Scientific, Fair Lawn, NJ) and frozen in 2-methylbutane submerged in liquid nitrogen. Tissues were cross-sectioned at 9 μm using a LEICA cryostat LC1900 sectioner, placed on Superfrost Plus microscopic slides (Fisher Scientific, Pittsburgh, PA), air-dried at room temperature, and frozen at -80°C until further use.

In the experiments using hematoxylin and eosin staining, the tissue slides were rehydrated in ethanol baths, immersed in Gill's Hematoxylin 3 and 1% eosin Y (Fisher Scientific, Fair Lawn, NJ), and then dehydrated in ethanol. Slides were mounted in permanent media and examined using a Nikon TE300 microscope with a SPOT camera attached (Diagnostic Instruments, Inc., Detroit, MI). For experiments using fluorescence staining, the tissue slides were permeabilized with 1:1 acetone:methanol and blocked with 0.1% BSA. For direct visualization of GFP staining, the slides were counterstained with DAPI (Molecular Probes, Portland, OR) and mounted with Vectashield (Vector Laboratories, Inc., Burlingame, CA). For staining with anti-HCMV antibody, the permeabilized slides were stained with anti-IE1 monoclonal antibody (Goodwin Institute of Cancer Research, Plantation, FL), and then with secondary anti-mouse IgG conjugated to FITC and/or Texas-Red (Vector Laboratories, Inc., Burlingame, CA), prior to counterstain with DAPI. Images were visualized on a Nikon PCM2000 confocal microscope system [46]. The monoclonal antibodies against cytokeratins K13 and K14 were purchased from United States Biological (Swampscott, MA).

### Western analysis

The tissues were either mock-infected or infected with 2 × 10^4 ^PFU of different HCMV strains and mutants, then incubated for 0–10 days. Viral proteins were isolated as described previously [47]. The polypeptides from cell lysates were separated on either SDS/7.5% polyacrylamide gels or SDS/9% polyacrylamide gels cross-linked with N,N"methylenebisacylamide, and transferred electrically to nitrocellulose membranes. We stained the membranes using the antibodies against HCMV proteins and human actin in the presence of a chemiluminescent substrate (Amersham Inc, Arlington Heights, IL), and analyzed the stained membranes with a STORM840 phosphorimager. Quantitation was performed in the linear range of protein detection [47]. The monoclonal antibodies c1202, c1203s, and c1207, which react with HCMV proteins UL44, IE1, and UL99; were purchased from Goodwin Institute for Cancer Research (Plantation, FL). The monoclonal antibody against human actin was purchased from Sigma Inc (St Louis, MO).

### Treatment of ganciclovir

Two different sets of experiments were carried out to study the effect of ganciclovir (GCV) [31,32] on HCMV replication in the oral tissues. First, the tissues were first pre-incubated with different concentrations (i.e. 10 μM and 100 μM) of GCV for 2 hours, and then incubated with the viral inoculum in the presence of GCV for 4 hours to initiate HCMV infection. In the second set of experiments, the tissues were incubated with viral inoculum for 4 hours in the absence of GCV, and then incubated in fresh media in the absence of GCV for additional 24 hours before adding different concentrations of GCV to the culture. The infected tissues were incubated in the GCV-containing media for different periods of time and harvested, and viral titers in these tissues were determined by plaque assays on HFFs.

### Growth kinetics of HCMV in cultured fibroblasts

Growth analyses of different HCMV strains and mutants in vitro in primary human foreskin fibroblasts (HFFs) were carried out as described previously [23]. Briefly, 1 × 10^6 ^human foreskin fibroblasts were infected at an MOI of 0.05 PFU per cell. The cells and media were harvested at 0, 2, 4, 7, 10 and 14 days post infection, and viral stocks were prepared by adding an equal volume of 10% skim milk, followed by sonication. The titers of the viral stocks were determined by plaque assays on HFFs in triplicates.

## Competing interests

The author(s) declare that they have no competing interests.

## Authors' contributions

All authors participated in conceiving, designing, and performing the experiments and analyses, and in writing the manuscript.
